# Features of structure, magnetic state and electrodynamic performance of SrFe_12−x_In_x_O_19_

**DOI:** 10.1038/s41598-021-97684-8

**Published:** 2021-09-15

**Authors:** V. A. Turchenko, S. V. Trukhanov, V. G. Kostishin, F. Damay, F. Porcher, D. S. Klygach, M. G. Vakhitov, D. Lyakhov, D. Michels, B. Bozzo, I. Fina, M. A. Almessiere, Y. Slimani, A. Baykal, D. Zhou, A. V. Trukhanov

**Affiliations:** 1 Joint Institute for Nuclear Research, 6 Joliot-Curie Str., 141980 Dubna, Russia; 2 South Ural State University, 76, Lenin Av., 454080 Chelyabinsk, Russia; 3 Donetsk Institute of Physics and Technology Named After O.O. Galkin of the NASU, 46 Nauki Av., Kiev, 03680 Ukraine; 4 SSPA “Scientific and Practical Materials Research Centre of NAS of Belarus”, 19 P. Brovki str., 220072 Minsk, Belarus; 5 National University of Science and Technology “MISiS”, Leninsky av., 4, Moscow, Russia 119049; 6 Laboratoire Leon Brillouin, UMR12 CEA-CNRS, Bât. 563 CEA Saclay, 91191 Gif sur Yvette Cedex, France; 7 Ural Federal University named after the First President of Russia B.N. Yeltsin, Yekaterinburg, Russia 620002; 8 Computer, Electrical and Mathematical Science and Engineering Division, 4700 King Abdullah University of Science and Technology, Thuwal, 23955-6900 Saudi Arabia; 9 Institut de Ciencia de Materials de Barcelona-CSIC, Campus de la UAB, 08193 Bellaterra, Barcelona Spain; 10 Present Address: Department of Biophysics, Institute for Research and Medical Consultations (IRMC), Imam Abdulrahman Bin Faisal University, P.O. Box 1982, Dammam, 31441 Saudi Arabia; 11 Department of Physics, College of Science, Imam Abdulrahman Bin Faisal University, P.O. Box 1982, Dammam, 31441 Saudi Arabia; 12 Department of Nanomedicine Research, Institute for Research and Medical Consultations (IRMC), Imam Abdulrahman Bin Faisal University, P.O. Box 1982, Dammam, 31441 Saudi Arabia; 13 Electronic Materials Research Laboratory, Key Laboratory of the Ministry of Education & International Center for Dielectric Research, School of Electronic Science and Engineering, Xi’an Jiaotong University, Xi’an, 710049 China

**Keywords:** Electronic devices, Magnetic materials

## Abstract

Indium-substituted strontium hexaferrites were prepared by the conventional solid-phase reaction method. Neutron diffraction patterns were obtained at room temperature and analyzed using the Rietveld methods. A linear dependence of the unit cell parameters is found. In^3+^ cations are located mainly in octahedral positions of 4f_VI_ and 12 k. The average crystallite size varies within 0.84–0.65 μm. With increasing substitution, the T_C_ Curie temperature decreases monotonically down to ~ 520 K. ZFC and FC measurements showed a frustrated state. Upon substitution, the average and maximum sizes of ferrimagnetic clusters change in the opposite direction. The M_r_ remanent magnetization decreases down to ~ 20.2 emu/g at room temperature. The M_s_ spontaneous magnetization and the k_eff_ effective magnetocrystalline anisotropy constant are determined. With increasing substitution, the maximum of the ε^/^ real part of permittivity decreases in magnitude from ~ 3.3 to ~ 1.9 and shifts towards low frequencies from ~ 45.5 GHz to ~ 37.4 GHz. The maximum of the tg(α) dielectric loss tangent decreases from ~ 1.0 to ~ 0.7 and shifts towards low frequencies from ~ 40.6 GHz to ~ 37.3 GHz. The low-frequency maximum of the μ^/^ real part of permeability decreases from ~ 1.8 to ~ 0.9 and slightly shifts towards high frequencies up to ~ 34.7 GHz. The maximum of the tg(δ) magnetic loss tangent decreases from ~ 0.7 to ~ 0.5 and shifts slightly towards low frequencies from ~ 40.5 GHz to ~ 37.7 GHz. The discussion of microwave properties is based on the saturation magnetization, natural ferromagnetic resonance and dielectric polarization types.

## Introduction

As a rule, the novel functional materials are very often created on the platform of complex oxides^1–3^. The main advantage of oxides is their chemical stability during operation of the material in an air atmosphere^4^. The development of new information technologies and wireless data transmission systems^5^ is based on materials with high microwave characteristics. Such materials are mainly composites of complex oxides of transition metals^6^. Since the microwave properties of a material are determined not only by the dielectric contribution, but also by the magnetic one, the use of magnetodielectrics is the most suitable solution to the problem^7^. Ferrites are the most typical representatives of complex transition metal oxides with optimal magnetodielectric properties^8^.

Ferrites of various types exist^9^. The most promising of them have spinel^10^ and magnetoplumbite structures^11^. Most often, ferrites are used to convert the energy of electromagnetic radiation^12^. The use of ferrites in microelectronic devices is explained by the high values of the Curie point and the spontaneous magnetic moment^13^. For hexaferrites with the M-type magnetoplumbite structure, high values of dielectric and magnetic permeability in the microwave region are especially distinguished^14^. One of the representatives of hard magnetic materials with a ferrimagnetic structure is M-type strontium hexaferrite SrFe_12_O_19_^15^. Due to the low cost of production, it is most often used as permanent magnets, microwave devices, and wireless communication components^16^.

The crystal structure of the M type SrFe_12_O_19_ hexaferrite, which is hexagonal with space group P6_3_/mmc (No 194), is designed from the 4 building blocks, namely S, S*, R, and R*. The asterisk indicates mirror symmetry about the (xy0) plane. The O^2−^ anions are closed packed with the Sr^2+^ and Fe^3+^ cations in the interstitial positons. There are 10 layers of the O^2−^ anions along the c axis and the Fe^3+^ catons are placed at 5 crystallographically nonequivalent positions: 2a, 4f_VI_ and 12 k octahedral ones, 4f_IV_ tetrahedral ones and 2b pentahedral (trigonal–bipyramidal) ones. The Fe^3+^ cations contribute to the magnetic moment in the next way: the 12 k, 2a, and 2b positions have spin up and 4f_IV_ and 4f_VI_ positions have spin down direction. The S = Fe_6_O_8_ and S* blocks are spinels with 2 O^2−^ layers and 6 Fe^3+^ cations. The 4 from these Fe^3+^ cations are placed in the octahedral positions with parallel spins. The other 2 Fe^3+^ cations are in tetrahedral positions with antiparallel spins to the octahedral positions. The hexagonal R = SrFe_6_O_11_ and R* blocks, consist of 3 O^2−^ anions layers with one of the O^2−^ anion replaced with an Sr^2+^ cation. The 6 Fe^3+^ cations are placed in each R block. The 5 Fe^3+^ cations are placed in octahedral positions. The 3 of them have spin up and 3 have spin down direction. The 1 Fe^3+^ cation is coordinated with the 5 O^2−^ anions and has spin up direction. The Fe^3+^ cations at the 2a positions are octahedrally coordinated with equal Fe–O lengths. The other octahedrally coordinated Fe^3+^ cations at 4f_VI_ and 12 k sites have different Fe–O lengths, from ~ 1.8 Å up to ~ 2.4 Å. The 12 Fe^3+^ cations of the formula unit, the Fe^3+^ cations at 4f_IV_ positions are tetrahedrally coordinated by the O^2−^ anions, while the Fe^3+^ cations at 2b positions are coordinated by the 5 O^2−^ anions. There are also short Fe–Fe lengths in the structure, and at 4f_VI_ positions these Fe–Fe lengths are ~ 2.7 Å. Such a complex unit cell is characterized by significant crystalline anisotropy, which is reflected in the ratio of lattice parameters c/a *≈* 3.96. So, each from the Fe^3+^ cations contributes 5 μ_B_ in the ground state, the total moment is (1*(2a) + 1*(2b) -2*(4f_V_) − 2*(4f_VI_) + 6*(12 k))*5 μ_B_ = 20 μ_B_ per formula unit^11,14^.

For the SrFe_12_O_19_ the saturation magnetic induction increased from ~ 291 G to ~ 300 G and the coercivity decreased from ~ 2.8 kOe to ~ 1.8 kOe by increasing the sintering temperature. The microwave absorption before sintering was below ~ 3 dB, but it reached ~ 6 dB at ~ 11.1 GHz after sintering. The reflection loss spectra increased by sintering due to the reduction of porosity and damping factor^17^. The magnetic permeability and magnetic loss measurements in the range 100 MHz to 1.5 GHz, reveals that 1.32 ≤ *μ*′ ≤ 1.68 for the permeability of BaFe_12_O_19_ and 1.16 < *μ*′ ≤ 1.88 for SrFe_12_O_19_. The BaFe_12_O_19_ presented lower loss ~ 4.10^−3^ at 1.5 GHz. The permittivity of BaFe_12_O_19_ and SrFe_12_O_19_ at 1.5 GHz are, respectively ~ 8.18 and ~ 8.19, and at 1 MHz are, respectively ~ 52.04 and ~ 19.09. The samples presented coercive field in the range of 3–5 kOe and remanence magnetization in the range of 33–36 emu/g^18^.

The substitution is also one of the ways to control the microwave properties of complex oxides. With increasing substitution for the SrFe_12−x_Ti_x/2_Zn_x/2_O_19_, the coercivity decreased from ~ 5640 Oe to ~ 1486 Oe while the maximum saturation magnetization was ~ 63 emu/g for the x = 0.5. It was established the excellent microwave absorption and the maximum loss was ~ 36.58 dB for the sample with x = 2.5^19^. One of the most interesting types of substitution is a diamagnetic homovalent substitution with the In^3+^ indium cations at which the charge state of the Fe^3+^ iron cations remains constant. The room temperature Mössbauer spectra confirmed that the In^3+^ cations preferentially occupy the 4f_VI_ octahedral positions in SrFe_12_O_19_ hexaferrite^20^. However, it was indications that the substitutes also enter the 2b trigonal–bipyramidal positions. The change in hyperfine magnetic field at the 12 k positions indicates the complex and competing behavior of the Fe^3+^–O^2−^–Fe^3+^ indirect superexchange interactions. The splitting of the 12 k magnetic hyperfine spectrum is not due simply to random disruptions of the indirect superexchange interactions.

Effects of nonmagnetic indium ion substitution on magnetic properties of M-type strontium hexaferrites SrFe_12−x_In_x_O_19_ (*x* = 0, 0.25, 0.5, 0.75, and 1) have been earlier studied by crystallographic and magnetic measurements^21^. Samples were prepared by citric auto-combustion method. It was shown that the samples were single phase with the P6_3_/mmc (No 194) space group and lattice constants linearly increased with substitution. It was established that In^3+^ cations mainly placed in 4f_VI_ positions. It was also shown that while the coercive force and the magnetic anisotropy constant decreased through the whole doping range, the saturation magnetization first increased until x = 0.5 and decreased thereafter^21^. Based on the calculation by the first-principles density functional theory of the substitution energy of In^3+^ cations in SrFe_12_O_19_ and the formation probability analysis, it was concluded that the substituted In^3+^ cations in SrFe_12−*x*_In_*x*_O_19_ occupy the 12 k, 4f_VI_, and 4f_IV_ positions. The positions occupation probabilities were used to calculate the magnetic properties of the substituted samples. It was found that in the case of SrFe_12−*x*_In_*x*_O_19_, saturation magnetization, magnetic anisotropy energy, and anisotropy field decrease with an increase of the substitution concentration^22^. To explain the appearance of the spontaneous polarization in the M-type hexaferrites the determination of the crystal structure features was realized in the centrosymmetric (No 194) and non-centrosymmetric (No 186) space groups^23,24^.

In our case the structure and microwave characteristics of the In^3+^ cations substituted strontium hexaferrites were thoroughly investigated. The powder neutron diffraction that allows collecting the crystal structure and magnetic ordering information was applied. The using of a wide range of experimental methods for studying the microwave performance of the obtained samples under different conditions allows to analyze the relationship between their charge and spin subsystems.

## Experimental procedure

All the ceramic SrFe_12−x_In_x_O_19_ (SFI-x) (x = 0.1; 0.3; 0.6 and 1.2) samples were obtained according to the method described in detail in the work^24^.

The homogeneity and chemical content of all the samples were checked by X-ray diffraction method on the diffractometer Empyrean (Panalytical, Malvern, United Kingdom) in Cu-radiation. Neutron diffraction studies of the ceramic samples were carried out at room temperature on G 4–1 and G 4–4 diffractometers with wavelength ~ 2.426 Å and ~ 1.9593 Å, respectively, in the experimental hall of the nuclear center in Laboratoire Leon Brillouin (LLB) (Saclay, France). The resolution functions for both G 4–1 and G 4–4 diffractometers were determined during separate experiments with NAC standard. The Rietveld analysis^25^ of neutron diffraction patterns as previously performed for similar chemical compounds^26^ was carried out by FullProf software package^27^.

The morphology of microstructure of ceramic samples were studied using a scanning electron microscope JSM-6490 LV (JEOL, USA). The microscope resolution reaches 3 nm with an accelerating voltage of the electron beam of 30 kV, the local areas of the sample increased up to × 20,000, the beam current was 200 nA.

The Curie temperature of samples was determined using the temperature dependence up to 800 K of their specific magnetization in field of 1 T^26^. The specific magnetization was measured on a MPMS XL SQUID (Quantum Design North America, USA) interferometer in the temperature range from 5 to 400 K^24^. The temperature dependences of the magnetization were measured in zero field cooled (ZFC) and field cooled (FC) modes in field of 50 Oe^28^. All the magnetic data were collected at warming.

The permittivity and permeability of the indium doped strontium hexaferrites bulk samples were extracted using the Nicholson-Ross-Weir algorithm from the S-parameters fixed as a frequency spectrum^29^. The measurements were realized in the frequency range of 32–50 GHz using the R&S ZVA 50 vector network analyzer (Rohde & Schwarz, Germany). When measuring S-parameters of the waveguide transmission line with the test material as a dielectric, a two-port TRL calibration was used.

The measuring line was used to determine the S-parameters. The samples for these experiments were prepared as powders. The waveguide WR-22 with dimensions 5.69 × 2.845 mm was used as a measuring line. The locking plates with electromagnetic parameters ε∼1.1 and μ∼1 were used to fix the investigated sample in the measuring line. The compensation of the influence of the locking plates on the measured parameters was carried out by calibrating the device.

## Result and discussion

### Crystal structure

**Figure 1:**
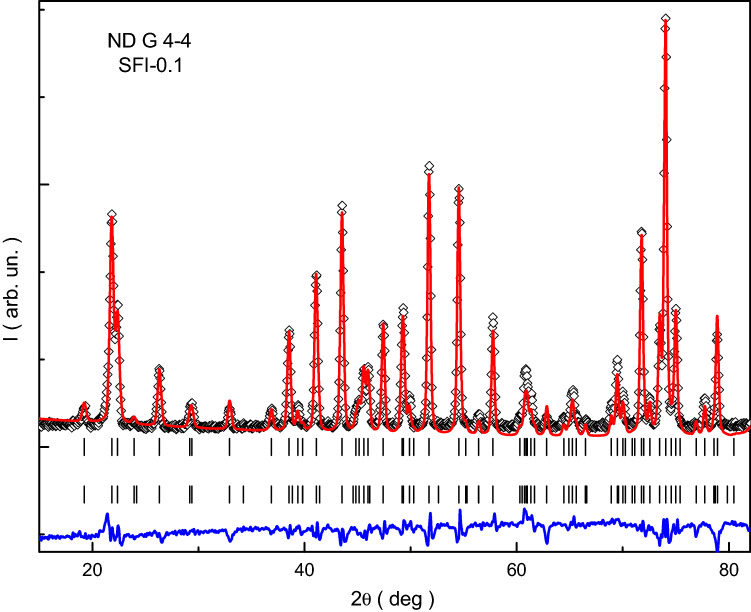
NPD pattern with Rietveld fitting lines at room temperature for the SFI-0.1 sample measured on diffractometer G 4–4.

To describe the features of crystal structure of the indium doped SFI-x strontium hexaferrites and their correlation with electromagnetic properties the powder neutron diffraction patterns at room temperature were collected, which are shown in Fig. 1. The numerical results of the refinement are shown in Table 1.

**Table 1:** Refined unit cell parameters, atomic positions and reliability factors for the *SrFe*_12*−x*_*In*_*x*_*O*_19_ (x = 0.1–1.2) samples obtained by the Rietveld method in framework of the *P*6_3_*/mmc* (No. 194) space group at room temperature. Fixed atomic positions for this case are: Sr (2d) (2/3, 1/3, 1/4), Fe1 (2a) (0, 0, 0); Fe2 (2b) (0, 0, 1/4); (Fe/In)3 (4*f*_*IV*_) (1/3, 2/3, z); (Fe/In)4 (4*f*_*V I*_) (1/3, 2/3, z); (Fe/In)5 (12 k) (x, 2x, z); O1 (4e) (0, 0, z); O2 (4f.) (1/3, 2/3, z); O3 (6 h) (x, 2x, 1/4); O4 (12 k) (x, 2x, z); O5 (12 k) (x, 2x, z).

Unit cell parameters and atoms	x			
	0.1	0.3	0.6	1.2
a, (Å)	5.8779 (3)	5.8930 (2)	5.9042 (3)	5.9148 (4)
c, (Å)	23.0424 (3)	23.0980 (5)	23.1495 (4)	23.2196 (4)
V, (Å^3^)	689.53 (3)	694.68 (3)	698.86 (4)	703.74 (4)
**Fe3/In (4*f***_***IV***_**)**				
z	0.0308 (5)	0.0315 (6)	0.0284 (5)	0.0225 (4)
**Fe4/In (4*f***_***VI***_**)**				
z	0.1931 (4)	0.1892 (5)	0.1892 (4)	0.1869 (3)
**Fe5/In (12* k*)**				
x	0.1849 (11)	0.1683 (19)	0.1684 (17)	0.1624 (3)
z	− 0.1087 (3)	− 0.1098 (2)	− 0.1097 (2)	− 0.1079 (4)
**O1 (4*e*)**				
z	0.1437 (6)	0.1539 (8)	0.1526 (7)	0.1512 (6)
**O2 (4f.)**				
z	− 0.0369 (5)	− 0.0501 (9)	− 0.0548 (8)	− 0.0618 (7)
**O3 (6* h*)**				
x	0.1709 (5)	0.1815 (45)	0.1798 (42)	0.1942 (3)
**O4 (12* k*)**				
x	0.1646 (3)	0.1587 (31)	0.1563 (28)	0.1606 (3)
z	0.0551 (2)	0.0553 (4)	0.0537 (4)	0.0545 (5)
**O5 (12* k*)**				
x	0.4939 (3)	0.5112 (32)	0.5059 (36)	0.5078 (3)
z	0.1532 (2)	0.1533 (4)	0.1515 (3)	0.1506 (2)
*R*_*wp*_, %	6.09	13.4	12.2	5.78
*R*_*exp*_, %	3.78	11.70	11.30	4.02
*R*_*B*_, %	4.46	17.8	12.5	5.15
*R*_*Mag*_, %	5.32	23.7	10.7	5.64
χ^2^	2.24	1.32	1.17	2.08

Figure 1 shows the experimental data in the form of individual intensity of the Bragg’s positions. This plot also shows the results of fitting in the form of continuous lines, one of which describes a continuous diffraction spectrum, and the second line gives an idea of the calculation error. The fitting was based on the Bragg’s positions shown in this plot, both crystal and magnetic contributions to the structure. So, the presented data fully reflects the necessary and sufficient set for the fitting and its results.

The classical values of unit cell parameters ionic coordinates and reliability factors were obtained^30^. As it was well seen the a parameter linearly increases with x from ~ 5.8786(2) Å for the x = 0.1 up to ~ 5.9159(3) Å for the x = 1.2. Such behavior of the parameters satisfies the Vegard’s low^31^. It is determined by the ionic radii of r(Fe^3+^) = 0.645 Å and r(In^3+^) = 0.800 Å^32^. The unit cell V volume continuously rises from ~ 689.64(2) Å^3^ for the x = 0.1 up to ~ 703.77(5) Å^3^ for the x = 1.2. It is established that the In^3+^ cations occupy mainly the octahedral 4f_VI_ and 12 k and tetrahedral 4f_IV_ positions. The χ^2^ satisfactory factor of fitting did not exceed ~ 2.38. At the structure fitting the results of^33^ were also taken into account.

The conclusion about the distribution of the substitutional In^3+^ cations over the sublattices was made based on the results of the refinement of the crystal structure by the Rietveld method. The result of fitting with the most optimal values of the reliability factors was used. When forming the model, data were used previously obtained by several groups of researchers^34–36^. The results obtained on the distribution of substituent indium cations are in satisfactory agreement with recent works^37–39^, which indicates the reliability of the model used.

### Microstructure

**Figure 2:**
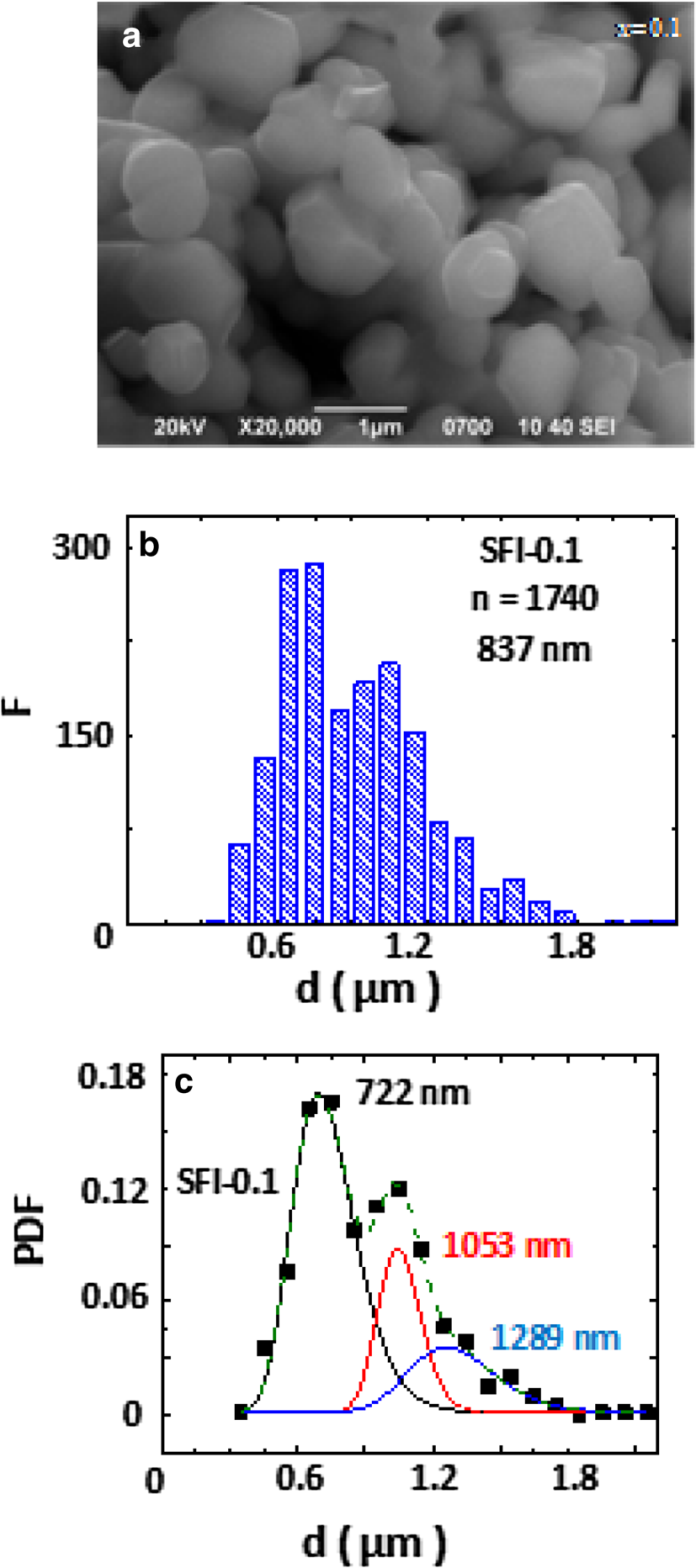
Surface morphology for the SFI-0.1 sample (**a**), the distribution histograms vs. particle diameter (**b**) and the approximation of the average probability density by the three-modal function of the lognormal distribution (**c**).

The surface form and numerical calculation results for the SFI-0.1 sample are demonstrated in Fig. 2a-c. As can be easy to see from SEM image, the sample has some porosity of *∼* 15%. This fact is explained by the insufficient annealing duration and weak preliminary compaction. The particles form is best described by the volumetric polyhedra. The size distribution histogram with the (n) particles number and their average particles size are also presented in Fig. 2b. The average particles size for all the samples is in the range 837–650 nm. It is interesting to note the formation of large crystallites from different particles with sizes exceeding ~ 1 µm for all the samples. The number of particles taken for size analysis was around 1500 for each sample.

The three- and bimodal functions were applied to describe the average particles size results and to receive the probability density function. For the SFI-0.1 sample the approximation of the average probability density by three-modal function of the lognormal distribution shows a good coincidence (see also Fig. 2c). The average particle sizes for each fraction of polydisperse samples were determined by the method of deconvolution of the lognormal function.

### Magnetic properties

**Figure 3:**
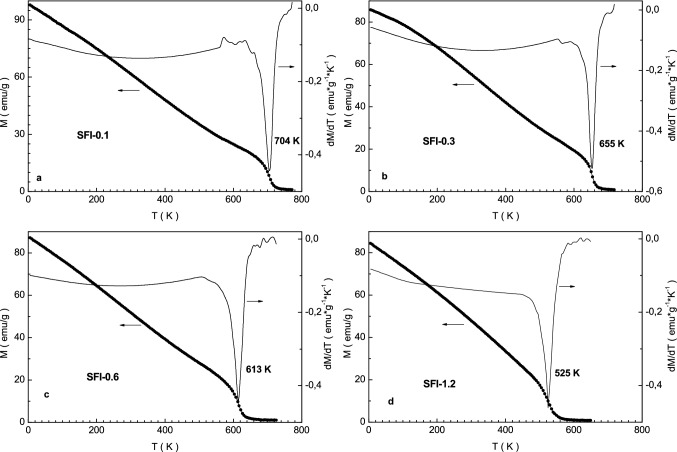
Graphs of magnetization (left axis) and magnetization derivative (right axis) vs. temperature in 1 T for the samples SFI-0.1 (**a**), SFI-0.3 (**b**), SFI-0.6 (**c**) and SFI-1.2 (**d**).

The temperature dependence of the specific magnetization in field of 1 T for the SFI-x solid solutions is shown in Fig. 3. Starting from low temperatures the signal continuously decreases. This fact is explained by acting of ever-increasing thermal energy. The latter disorders local spins and magnetization falls. The T_C_ Curie temperature of the compounds monotonically decreases from ~ 730 K down to ~ 520 K with increasing the x (see insert in Fig. 3). Such behavior is a result of frustration of the magnetic ordering because of breakdown of long-range Fe^3+^ –O^2−^–Fe^3+^ indirect superexchange interactions by diamagnetic substitution that is consistent with analogous data earlier obtained for the indium doped barium hexaferrites^40^.

**Figure 4:**
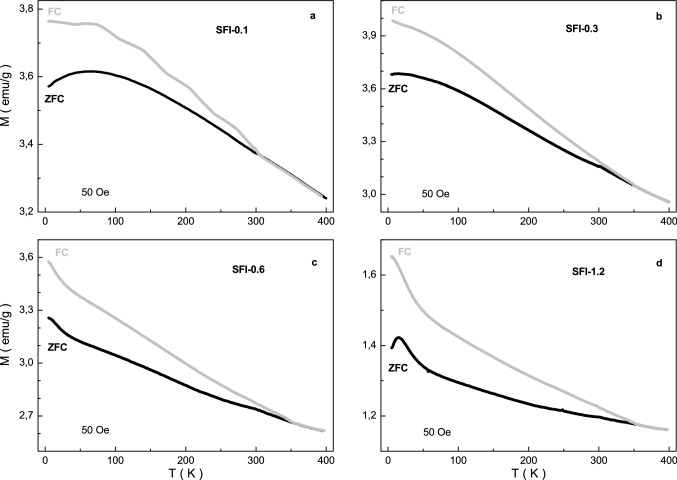
Graph of ZFC (black line) and FC (grey line) magnetization vs. temperature in a field of 50 Oe for the samples SFI-0.1 (**a**), SFI-0.3 (**b**), SFI-0.6 (**c**) and SFI-1.2 (**d**).

The temperature dependence of the magnetization for the investigated compounds was also collected in ZFC and FCW modes. They are shown in Fig. 4. It is well seen that the ZFC and FC curves are strong different below some temperature and the ZFC curve has local maximum. With increase the x the temperature of local maximum decreases from ~ 65 K down to ~ 13 K while the temperature of divergence of ZFC and FC curves increases from ~ 327 K up to ~ 362 K.

The divergence between ZFC and FC curves are observed for all the compositions above the room temperature. It is should be note that similar divergence between ZFC and FC curves was previously observed for different compounds and magnetic fine particles^41–44^. The existence of the ZFC and FC curves divergence below a certain T_div_ temperature is a characteristic of a frustrated magnetic system^45^. Decreasing the temperature without external magnetic field leads to freezing of local moments at a certain T_f_ freezing temperature. The preferred orientation is absent and all the moments are randomly frozen relative to each other. As a result of increasing the magnetic anisotropy a decrease in the magnetization of the sample is observed^46^.

At the cooling of the sample in the magnetic field the moment direction is determined by the competition between the energy of magnetic crystallographic anisotropy and Zeeman energy^41^. In large fields the moments in all crystallites are ordered parallel with the field and the magnetic texture ensures the presence of residual magnetization at low temperatures^47^. The T_f_ freezing temperature below which the energy of thermal fluctuations is significantly lower than the energy of magnetic anisotropy, can be found from the Bean-Livingston equation^48^.

Such distinction of the ZFC and FC curves for all the samples may be explained by the high stiffness and anisotropy of the Fe^3+^–O^2−^–Fe^3+^ exchange interactions^49^. As it well known the T_f_ freezing temperature determines the average size of a magnetically ordered inclusion, which can be a magnetically ordered cluster, in a magnetically disordered matrix or just a fine particle in powder. The T_div_ divergence temperature determines the maximum size of the magnetically ordered inclusion.

**Figure 5:**
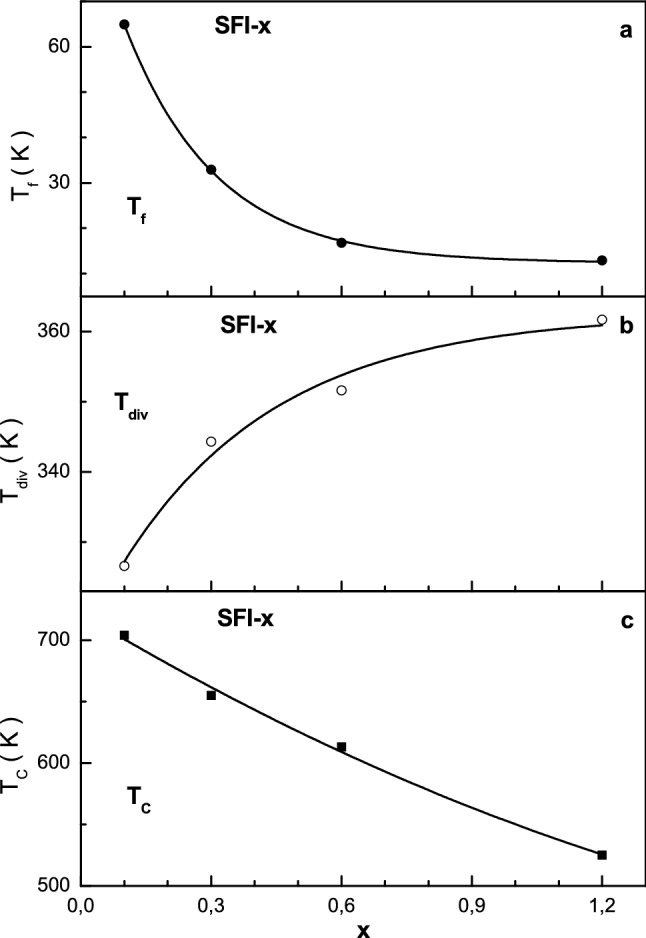
Graph of the T_f_ freezing (**a**) and T_div_ divergence (**b**) and T_C_ Curie (**c**) temperature vs. x for the SFI-x samples.

The concentration behavior of the critical T_f_, T_div_ and T_C_ temperatures is presented in Fig. 5 and may be explained by a change in the average and maximum sizes of magnetically ordered inclusion. The average size monotonically decreases with a diminishing rate of -47.3 K/x. That means that the substitution leads to the fragmentation of magnetically ordered inclusions. The maximum size monotonically increases with a diminishing rate of 31.9 K/x. That means that the substitution leads to the overgrowth of large magnetically ordered inclusions^50^.

**Figure 6:**
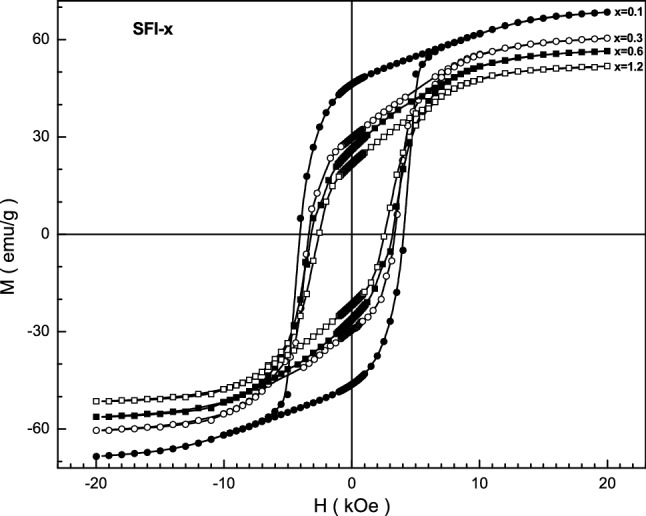
Graph of magnetization vs. field for the SFI-x samples with x = 0.1 (full circles), 0.3 (open circles), 0.6 (full rectangles) and 1.2 (open rectangles) at 300 K.

The field dependences of the magnetization for the investigated SFI-x samples measured at temperatures 300 K are shown in Fig. 6. The values of the M_r_ residual magnetization measured after switching off the external magnetic field, and the squareness ratio of the hysteresis loop SQR = M_r_/M_s_ as well as the values of the H_c_ coercive field are shown in Table 2.

**Table 2:** The M_r_ residual magnetization, M_s_ saturation magnetization, SQR squareness ratio, k_eff_ effective constant of magnetocrystalline anisotropy, H_a_ anisotropy field and H_c_ coercive field at room temperature.

x	M_r_, emu/g	M_s_, emu/g	SQR	K_eff_, *10^6^ erg/cm^3^	H_a_*10^4^, Oe	H_c_, Oe
0.0	56.23	68.32	0.81	3.021	1.634	6532
0.1	46.32	59.59	0.78	2.974	1.378	4055
0.3	29.72	51.83	0.57	2.596	1.268	3371
0.6	26.18	48.57	0.54	2.355	1.244	3174
1.2	21.72	44.03	0.49	1.503	1.191	2544

With increasing the x the M_s_ saturation magnetization, M_r_ residual magnetization and H_c_ coercivity decrease. This is explained by the diamagnetic dilution of magnetic sublattice and increasing the non-collinearity angle^51^. The diamagnetic In^3+^ indium cations have a similar effect, the increase their amount breaks the Fe^3+^ –O^2−^–Fe^3+^ indirect superexchange interactions of neighboring Fe^3+^ iron cations and conducts to violation of the spin collinearity of the Fe^3+^ cations. As a result, the value of the M_r_ residual magnetization decreases from ~ 55.6 emu/g down to ~ 20.2 emu/g at room temperature as x is increased from 0.1 up to 1.2.

Figure 6 shows the behavior of the H_c_ coercive force of the SFI-x samples. It is well seen that for the SFI-x composition with x = 0.1 has value of the H_c_ coercive force of ∼ 4100 Oe whereas the x = 1.2 has the H_c_ of ~ 2520 Oe.

The Law of Approach of Saturation has been used to estimate the k_eff_ effective magnetocrystalline anisotropy constant of In^3+^ substituted strontium hexaferrite^52–54^:

1 $$M\left(H\right)={M}_{S}\left(1-\frac{B}{{H}^{2}}\right)+{\chi }_{d}H$$

where *M*_*s*_, *B* and χ_d_ are refined parameters: *M*_*s*_ is the saturation magnetization, χ_d_ is the susceptibility of the para-process. The rotation of magnetization by a strong magnetic field is associated with the term of *B/H*^2^, while the terms with *H*^*−n*^ (where n > 2) are usually neglected. In accordance with the Stoner–Wohlfarth theory^55^, the homogeneous magnetization of the sample is achieved by the strong interatomic exchange interaction, which exceeds the anisotropy energy. Therefore, the magnetization reversal process of a crystallite was carried out due to the rotation of the magnetization vector as well as by changing the anisotropy energy and Zeeman energy in an external magnetic field, while the exchange energy does not change^56^. In this case, the anisotropy field of the material increases with increasing the magnetic anisotropy constant and decreases with increasing saturation magnetization according to the equation^57^:

2 $${H}_{a}(T)=\frac{2{K}_{1}(T)}{{M}_{S}(T)}$$

where *M*_*s*_ is the saturation magnetization; *K*_1_ is the magnetocrystalline anisotropy constant. Therefore, the temperature dependences of the coercivity should be determined by competition between the constant of magnetic anisotropy and saturation magnetization. In this case, a decrease in H_a_ with decreasing temperature can be explained by the fact that the saturation magnetization increases faster than the effective constant of magnetic anisotropy. The presence of maximum on the ZFC curve for the investigated samples (see Fig. 4) is the result of the sum of narrow maxima for particles with certain sizes that indicates the presence of size distribution of particles. Therefore, the broadening of maximum is determined by the contribution of the volume fractions of particles with a certain size. Using the T_f_ freezing or T_div_ divergence temperatures it is possible to calculate the average or maximum size of magnetic clusters. In this case, the volume of magnetic regions make up (1.7–6.3) × 10^4^ Å^3^ in the case of the freezing T_f_ temperature and (3.4–8.0) × 10^5^ Å^3^ for the divergence T_div_ temperature, respectively. As a result, the linear size of the magnetic inclusions can achieve up to 32–49 Å and 87–115 Å, respectively.

### Microwave properties

**Figure 7:**
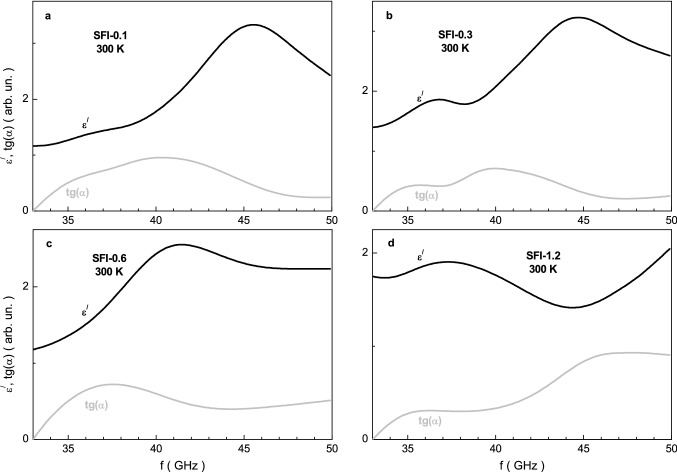
Graph of the ε^/^ real part of permittivity (black line) and tg(α) loss tangent (grey line) vs. frequency at room temperature for the SFI-x samples with x = 0.1 (**a**), 0.3 (**b**), 0.6 (**c**) and 1.2 (**d**).

Figure 7 shows the frequency dependence of the real part ε^/^ of permittivity and tg(α) dielectric loss tangent for the investigated samples at room temperature. These curves are characterized by local maximum in frequency range of 35–48 GHz. The resonance frequency constantly decreases with increasing the x. The amplitude of the resonance peak of the ε^/^ firstly increases up to ~ 3.3 at ~ 45.5 GHz for the x = 0.1 and then constantly decreases down to x = 1.2. The smallest value of ε^/^ local maximum is ~ 1.9 at 37.3 GHz for the x = 1.2. The dipole polarization, which determines the value of the real part ε^/^ of the permittivity, is associated with the orientation of the dipoles in the external electric field and is due to losses in overcoming the bond strengths.

It is well known that the complex $\dot{\varepsilon }$ permittivity and $\dot{\mu }$ permeability can be found using the S-parameters by the Nicolson–Ross–Weir method^58^ as:

3 $$\dot{\varepsilon } = iclg\left( {\frac{7}{3}} \right)\left( {1 - \mathop {S_{{11}} }\limits^{.} } \right){\text{ln}}\left( {\mathop {S_{{21}} }\limits^{.} } \right)/\upomega{\text{l}}Z_{B} \left( {1 + \mathop {S_{{11}} }\limits^{.} } \right)$$

and

4 $${\dot{\upmu }} = icZ_{B} \left( {1 + \mathop {S_{{11}} }\limits^{.} } \right){\text{ln}}\left( {\mathop {S_{{21}} }\limits^{.} } \right)/60\upomega{\text{l}}lg\left( {\frac{7}{3}} \right)\left( {1 - \mathop {S_{{11}} }\limits^{.} } \right)$$

where ${\mathop {S_{{11}} }\limits^{.} }$ is the reflection coefficient; ${\mathop {S_{{21}} }\limits^{.} }$ is the transmission coefficient; c is the speed of light; l—the length of the investigated sample; Z_B_—the transmission line wave resistance; ω—the cyclic frequency.

It is well seen that the tg(α) curves also have local maximum in investigated frequency range. The resonant frequency of the tg(α) firstly decreases down to ~ 38.4 GHz with increasing the x up to 0.6 and then increases above 45 GHz. The amplitude of the tg(α) resonant peak firstly increases up to ~ 1.0 at ~ 40.6 GHz as the x achieves the 0.1. Then it decreases down to ~ 0.7 at ~ 37.3 GHz with increasing the x up to 0.6. And finally it increases up to ~ 1.0 at ~ 46.7 GHz for the x > 1.2.

The main types of losses of incident electromagnetic radiation are due to the peculiarities of the microstructure and shape of the material, electromagnetic parameters, as well as impedance matching. Microwave absorption is determined by the contribution of dielectric and magnetic losses. Dielectric losses are due to conduction losses, i.e. eddy currents, and losses of dipole and interface polarization, while magnetic losses depend on the resonance of domain walls and natural ferromagnetic resonance. The ε^/^ real part of permittivity determines the loss of conductivity, and its ε^//^ imaginary part determines the loss of the dipole and interface polarization. The μ^/^ real part of permeability determines the loss of susceptibility of the spin subsystem to an external alternating magnetic field of electromagnetic radiation, and its μ^//^ imaginary part determines the losses from resonance of domain walls and natural ferromagnetic resonance.

So, the dielectric loss is determined by the ε^//^ imaginary part of permittivity, at lower frequencies, when dipole polarization prevails, this loss is very large, since the value of ε^//^ in the sample is comparable in magnitude with ε^/^. With increasing frequency, the dipole polarization goes over to the ion-relaxation polarization. With this transition, the loss decreases, which is expressed in a decrease in the absolute value of tg(α) (Fig. 7).

A decrease in the value of the ε^/^ real part of permittivity in the sample in the frequencies below 36 GHz is also due to the transition from dipole polarization to ion-relaxation polarization. This transition from one type of polarization to another is clearly visible because the maximum of the real part ε^/^ of the permittivity shifts to the low-frequency region, while the value of the maximum itself decreases.

Since the loss in all substances decreases at the transition from orientation polarization to ion-relaxation one, the value of the ε^//^ imaginary part of permittivity generally decrease in all samples above some critical frequency value (Fig. 7). When an electromagnetic wave acts on a substance, an ion with a larger mass is less displaced from its equilibrium position in the crystal lattice, and therefore dielectric losses decrease. Therefore, with increasing the indium substitution of strontium hexaferrites the loss value decreases, in our case. This indirectly confirms the above conclusion about the observed transition from orientation to ion-relaxation polarization. With increasing frequency, the orientation of the dipoles does not have time to change in accordance with the frequency of the applied field. As a result, the dipole polarization transforms into ion-relaxation polarization. Ion-relaxation polarization is caused by the displacement of ions at the sites of the crystal lattice, or located between sites^59^.

**Figure 8:**
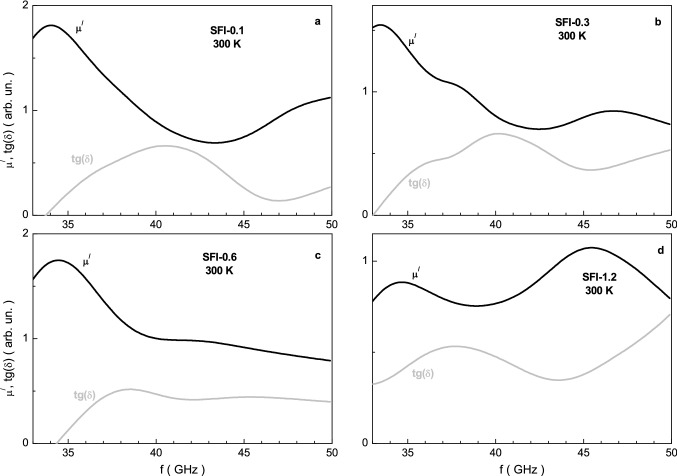
Graph of the μ^/^ real part of permeability (black line) and tg(δ) loss tangent (grey line) vs. frequency at room temperature for the SFI-x samples with x = 0.1 (**a**), 0.3 (**b**), 0.6 (**c**) and 1.2 (**d**).

Figure 8 demonstrates the frequency spectra of the μ^/^ real part of permeability and tg(δ) magnetic loss tangent for the investigated samples. The permeability has some features in the same frequency range as the permittivity. As a tendency, the value of the real part μ^/^ of the permeability decreases with increasing frequency. However, for some samples such as x = 0, 0.3 and 1.2, a local maximum is observed, while for other samples only a tendency towards a maximum. For samples with x = 0.0 and 0.1, the local maximum of μ^/^ shifts to the frequency range well above 50 GHz. Value of local maximum of ~ 1.5 at ~ 36.2 GHz is observed for the sample with x = 0. For other samples the low-frequency maximum shifts well below 35 GHz. However high-frequency maximum is fixed at ~ 46.7 GHz and ~ 45.5 GHZ in magnitude equal to ~ 0.9 and 1.1 for the samples with x = 0.3 and 1.2, respectively.

The value of the tg(δ) magnetic loss tangent decreases as a tendency with increasing frequency. The maximum of the tg(δ) is indicated for all the samples in range of 35–40 GHz. The high-frequency maximum is absent. The minimum of the tg(δ) is visible in vicinity of 45 GHz.

In a sample, each ion has a magnetic moment due to the geometry of unpaired electron clouds. Some ions have a compensated magnetic moment, i.e. they are diamagnetic. In a magnetic field inside each ion, weak induction currents of electron clouds are generated according to Faraday’s law of electromagnetic induction. They cause a periodic change in the orientation of the magnetic moment on average along the field. This is the so-called precession of the moment. According to Lenz’s law, the magnetic flux generated by induction currents is opposite to the external magnetic field. All the investigated samples behave like ferromagnets, for such materials μ ≥ 1^60^. The change in the magnetic permeability with frequency can be explained using Larmor’s theorem^61^. At rest, the samples have their own magnetic moment, which has a finite value, and has a significant impact on the overall value of the permeability. When the sample is placed in an external alternating magnetic field, according to Larmor’s theorem, the precession of the magnetic moment of the electrons begins. The energy spent on moment precession increases significantly at resonance and represents magnetic losses, i.e. the imaginary part μ^//^ of the permeability. Diamagnetic In^3+^ indium cations have zero moment and their contribution in magnetic resonance and the imaginary part μ^//^ of the permeability will decrease with increasing substitution^62^.

With an increase in in frequency and the substitution concentration by the In^3+^ indium cations, which have a large number of electrons, the intensity of the precession of the magnetic moment of electrons increases^63–65^ and, as a consequence, an increase in the real part μ^/^ of the permeability is observed. Due to the small induced magnetic moment of the In^3+^ indium cation itself, with an increase in its concentration^66–68^, this insignificantly affects the total value of μ^/^. An increase in the absolute value of the imaginary part μ^//^ of the permeability (Fig. 8) with an increase in the substitution concentration^69^ can be explained by an increase in the precession frequency of the magnetic moments and an increase in the energy expended to maintain the precession, i.e. magnetic losses.

## Conclusions

The SFI-x (x = 0.1; 0.3; 0.6 and 1.2) solid solutions were obtained by conventional solid-state reaction method from required oxides and carbonate. The Rietveld analysis of neutron diffraction patterns was realized. The linear increasing behavior of the unit cell parameters in accordance with the Vegard’s low was established which was explained by the radii of the substituted and substituting cations. It is revealed that the In^3+^ indium cations occupy mainly the octahedral 4f_VI_ and 12 k and tetrahedral 4f_IV_ positions. The maximum porosity of the samples reached *∼* 15%. The average size of particles is in the range 0.84–0.65 nm. The Curie temperature of the compounds monotonically decreases from down to ~ 520 K with increasing the x. Such behavior is a result of frustration of the magnetic ordering because of breakdown of long-range Fe^3+^ –O^2−^–Fe^3+^ indirect superexchange interactions. The frustrated magnetic state was detected from ZFC and FC magnetization measurements. The average size of magnetically ordered inclusions monotonically decreases with a diminishing rate of -47.3 K/x that means that the substitution leads to their fragmentation. The maximum size magnetically ordered inclusions monotonically increases with a diminishing rate of 31.9 K/x that means that the substitution leads to their overgrowth. The value of the M_r_ residual magnetization decreases to ~ 20.2 emu/g at room temperature. The M_s_ spontaneous magnetization and k_eff_ effective magnetocrystalline anisotropy constant were determined by the Law of Approach of Saturation. The amplitude of the resonance peak for the real part ε^/^ of the permittivity firstly increases up to ~ 3.3 at ~ 45.5 GHz for the x = 0.1 and then constantly decreases down to ~ 1.9 for the x = 1.2. The ε^/^ smallest value of local maximum is ~ 1.9 at 37.3 GHz for the x = 1.2. The maximum of the tg(α) dielectric loss tangent decreases from ~ 1.0 to ~ 0.7 and shifts towards low frequencies from ~ 40.6 GHz to ~ 37.3 GHz. The low-frequency maximum of the μ^/^ real part of permeability decreases from ~ 1.8 to ~ 0.9 and slightly shifts towards high frequencies up to ~ 34.7 GHz. The maximum of the tg(δ) magnetic loss tangent decreases from ~ 0.7 to ~ 0.5 and shifts slightly towards low frequencies from ~ 40.5 GHz to ~ 37.7 GHz. The maximum of the tg(δ) magnetic loss tangent decreases from ~ 0.7 to ~ 0.5 and shifts slightly towards low frequencies from ~ 40.5 GHz to ~ 37.7 GHz. The consistent change in the magnetic and microwave properties of the SFI-x hexaferrites is interpreted on the basis of a regular change in the saturation magnetization depending on the diamagnetic substitution by the In^3+^ indium cations and on the features of the transition in the type of polarization and natural ferromagnetic resonance.
